# Graded Multidimensional Clinical and Radiologic Variation in Patients With Alzheimer Disease and Posterior Cortical Atrophy

**DOI:** 10.1212/WNL.0000000000209679

**Published:** 2024-07-23

**Authors:** Ruth U. Ingram, Dilek Ocal, Ajay Halai, Gorana Pobric, David M. Cash, Sebastian Crutch, Keir X. Yong, Matthew A. Lambon Ralph

**Affiliations:** From the Division of Psychology and Mental Health (R.U.I., G.P.), University of Manchester; Dementia Research Centre (D.O., D.M.C., S.C., K.X.Y.), UCL Institute of Neurology, London; and MRC Cognition and Brain Sciences Unit (A.H., M.A.L.R.), University of Cambridge, United Kingdom.

## Abstract

**Background and Objectives:**

Alzheimer disease (AD) spans heterogeneous typical and atypical phenotypes. Posterior cortical atrophy (PCA) is a striking example, characterized by prominent impairment in visual and other posterior functions in contrast to typical, amnestic AD. The primary study objective was to establish how the similarities and differences of cognition and brain volumes within AD and PCA (and by extension other AD variants) can be conceptualized as systematic variations across a transdiagnostic, graded multidimensional space.

**Methods:**

This was a cross-sectional, single-center, observational, cohort study performed at the National Hospital for Neurology & Neurosurgery, London, United Kingdom. Data were collected from a cohort of patients with PCA and AD, matched for age, disease duration, and Mini-Mental State Examination (MMSE) scores. There were 2 sets of outcome measures: (1) scores on a neuropsychological battery containing 22 tests spanning visuoperceptual and visuospatial processing, episodic memory, language, executive functions, calculation, and visuospatial processing and (2) measures extracted from high-resolution T1-weighted volumetric MRI scans. Principal component analysis was used to extract the transdiagnostic dimensions of phenotypical variation from the detailed neuropsychological data. Voxel-based morphometry was used to examine associations between the PCA-derived clinical phenotypes and the structural measures.

**Results:**

We enrolled 93 participants with PCA (mean: age = 59.9 years, MMSE = 21.2; 59/93 female) and 58 AD participants (mean: age = 57.1 years, MMSE = 19.7; 22/58 female). The principal component analysis for PCA (sample adequacy confirmed: Kaiser-Meyer-Olkin = 0.865) extracted 3 dimensions accounting for 61.0% of variance in patients' performance, reflecting general cognitive impairment, visuoperceptual deficits, and visuospatial impairments. Plotting AD cases into the PCA-derived multidimensional space, and vice versa, revealed graded, overlapping variations between cases along these dimensions, with no evidence for categorical-like patient clustering. Similarly, the relationship between brain volumes and scores on the extracted dimensions was overlapping for PCA and AD cases.

**Discussion:**

These results provide evidence supporting a reconceptualization of clinical and radiologic variation in these heterogenous AD phenotypes as being along shared phenotypic continua spanning PCA and AD, arising from systematic graded variations within a transdiagnostic, multidimensional neurocognitive geometry.

## Introduction

Alzheimer disease (AD) generates heterogeneous amnestic (typical) and nonamnestic (atypical) phenotypes,^1,2^ including visual, logopenic, behavioral, and dysexecutive presentations.^3^ Posterior cortical atrophy (PCA) includes symptoms of space and object perception deficits, constructional apraxia, environmental agnosia, and alexia^4^ and is sometimes considered a “visual-spatial AD”^5^ variant. However, considering PCA as categorically distinct from AD, that is, adopting categorical classifications of AD variants, does not fully capture the graded variation within and between variants or mixed phenotypes.^2,4,6^ This presents challenges for diagnosing AD variants; selecting appropriate therapeutics and rehabilitation pathways; and research recruitment.^5,6^ The current study used deeply phenotyped neuropsychological and neuroimaging data in AD and PCA to explore graded patient variations, rather than categorical classifications, to establish and map the neuropsychological and neuroimaging dimensions that underpin transdiagnostic (i.e., encompassing both diagnostic groups) variations in these patients.

Previous comparative studies have shown PCA and amnestic AD differ in key cognitive and visual domains (e.g., delayed auditory/verbal memory worse in amnestic AD), but not significantly in others (e.g., working memory, language, ideomotor praxis). For example, although dorsal/spatial and ventral/perceptual subtypes of PCA have been proposed,^7^ impairments in other cognitive domains are also documented, such as linguistic impairments comparable with logopenic progressive aphasia (“language-variant AD”)^8^ and verbal short-term memory deficits found in some PCA cases, reminiscent of language-led AD.^9^ Furthermore, in amnestic (typical) AD, impairments in nonamnestic (atypical) domains including visuospatial processing have been found.^2,10^ These findings highlight the potential for graded, overlapping cognitive variation within and between PCA and AD, which may have been missed in many studies to date that use categorical classification systems to define groups.^9,11^ This gap can be addressed by using approaches that allow reconceptualizing of proposed variants/subtypes of patients as occupying subregions of a graded multidimensional space, with fuzzy boundaries between “groups”^2,10-12^ rather than discrete categorical classifications. Such approaches have been successfully applied to poststroke aphasia,^13^ primary progressive aphasia,^14^ semantic dementia,^15^ frontotemporal lobar degeneration,^16^ and logopenic progressive aphasia.^17^ This study therefore aimed to address this gap in AD and PCA by using an approach which (1) situates participants with amnestic AD and PCA in the same graded multidimensional space, rather than using contrastive group-level statistical comparisons, to better capture the patterns of overlapping and/or nonoverlapping cognitive performance, and then (2) relates the transdiagnostic phenotype dimensions to the pattern of atrophy across the whole brain, to understand how shared cognitive variation may reflect common atrophy patterns.

Using this approach, we hypothesized that we would find: (1) in AD, a dimension capturing graded variation in cognitive impairments characteristic of amnestic AD and a dimension capturing graded variation in visuospatial impairment (because this is commonly impaired in typical AD and thus included in global dementia measures such as the Mini-Mental State Examination [MMSE] and Addenbrooke's Cognitive Examination-Revised); (2) in PCA, dimensions capturing graded variation in visuospatial and visuoperceptual impairments (given the proposed dorsal/spatial and ventral/perceptual subtypes^7^) and a dimension capturing nonvisual, cognitive impairments too^9^; and (3) neural correlates for these extracted dimensions which reflect previous evidence of brain-behavior relationships in these patient groups, for example, occipitoparietal and occipitotemporal cortex for visuospatial and visuoperceptual dimensions, respectively,^18^ medial temporal lobe structures like entorhinal cortex^19^ plus interior parietal and lateral temporal cortices for dimensions capturing diverse, nonvisual impairments. Finally, given the prior evidence for overlapping phenotypic presentations within and between PCA and “typical” AD, we hypothesized that there would be overlapping graded variation in PCA and AD on these extracted dimensions and that this shared cognitive variation might be reflected by common atrophy patterns in these patient groups. Specifically, our hypotheses were explored through the application of principal component analysis to a detailed neuropsychological database followed by grey matter (GM) voxel-based morphometry (VBM), allowing a data-driven exploration of (1) the presence and cognitive nature of phenotypic continua in each group and (2) the extent of intragroup and intergroup graded variation in cognition and GM volume in the multidimensional space defined by these dimensions.

## Methods

### Study Population

All participants were recruited at a specialist center, the Dementia Research Centre at the National Hospital for Neurology and Neurosurgery, London, United Kingdom. All participants in this study were first interviewed on their history of behavioral, neuropsychiatric, dementia-related and non–dementia-related neurologic symptoms. Participants were then identified based on the interview and documentations related to their diagnosis, such as clinical letters and summaries of their medical and symptom history. All participants with PCA met consensus criteria for PCA-pure^4^ and clinical criteria of Tang-Wai et al.^20^ and Mendez et al.^21^ based on available information at baseline and expert retrospective clinical review. Participants with PCA were excluded from this study if there was evidence of non-AD dementia (i.e., dementia with Lewy bodies or corticobasal degeneration), including CSF/amyloid-PET incompatible with underlying AD and/or clinical features of early visual hallucinations, pyramidal signs, reduplicative phenomena, parkinsonism, alien limb syndrome, asymmetric dystonia and myoclonus, and ataxia. All participants with AD met the National Institute of Neurological and Communicative Disorders and Stroke and the Alzheimer Disease and Related Disorders Association criteria for probable AD with recently proposed revisions.^22^ Participants with AD were excluded if they showed a nonamnestic presentation consistent with the diagnostic criteria for atypical AD (PCA, logopenic progressive aphasia, corticobasal syndrome, or behavioral/dysexecutive AD). Consequently, this group consisted of participants with amnestic-led AD presentations (demographic details are shown in Table 1).

All available molecular or pathologic evidence (34 PCA; 39 AD) supported underlying AD pathology (63 had a CSF profile compatible with AD); 3 had positive amyloid PET scans, and 11 had autopsy-proven AD. Patients with biomarker evidence of AD pathology met the International Working Group-2 criteria of McKhann et al.^22^ for probable AD with high biomarker probability of AD aetiology.^23^

The PCA and AD cases have been included in previous publications.^12,19,24^ All patients provided informed consent under approval from NRES Committee London, Queen Square.

### Neuropsychological Assessments

Both groups completed the same neuropsychological battery, thus allowing direct comparisons. The neuropsychological assessments were completed typically on the same day as the neuroimaging scan, or where this was not possible, the scan and neuropsychological assessments took place within 3–6 months of each other. The tests included in the principal component analysis are shown in Table 2 and most are described in the study by Lehmann et al.,^24^ with the addition of letter “A” Cancellation,^25^ recognition memory for faces,^26^ and tests of early visual processing. The latter included hue discrimination,^27^ shape discrimination,^28^ figure/ground separation (Visual Object and Space Perception [VOSP] battery^29^), and crowding. Assessments measuring time to complete or number of errors, where a lower value indicates less impaired performance, were inverted so that lower values across all tests indicated worse performance. Significant differences between diagnostic groups on each neuropsychological test were assessed through independent *t* tests.

**Table 1:** Demographic Details for Each Diagnostic Group

Diagnosis	Total N (F)	Age (y)	Symptom duration (y)	MMSE	TIV (mm^3^)
AD	58 (22)	57.1 (6.4)	6.2 (3.0)	19.7 (4.9)	1,422.7 (134.1)
PCA	93 (59)	59.9 (8.1)	5.2 (2.6)	21.2 (5.1)	1,439.1 (158.3)

Abbreviations: AD = Alzheimer disease; MMSE = Mini-Mental State Examination; PCA = posterior cortical atrophy; TIV = total intracranial volume.

Age, symptom duration, MMSE score, and TIV are presented as mean (SD). The total sample size per group is given in “Total N” with the number of women in the group given in brackets (F). The sample size for TIV is 62 PCA, 9 AD.

### Cognitive Analysis

All raw cognitive scores were converted to percentages. For time-based measures without a fixed maximum score (letter “A” cancellation [time]; Crowding [time]; VOSP dot count [time]), scores were converted to a percentage of the maximum time taken within each cohort. The adequacy of the sample size for each principal component analysis was determined using the Kaiser-Meyer-Olkin measure of sampling adequacy and Bartlett's test of sphericity.

#### Imputation and Component Selection

To retain as much information (patients and tests) as possible, missing data were imputed using probabilistic principal component analysis,^30^ which was also used to select the optimal number of components for subsequent principal component analysis using the imputed dataset (as described in Ingram et al.,^14^ see eMethods). The subsequent principal component analyses were also run on a version of the dataset with missing data more strictly removed (see eMethods).

#### Principal Component Analysis

We applied separate principal component analyses to the AD and PCA cohorts to establish the multidimensional space of each presentation independently (this avoids the possible danger of creating false overlaps by fusing the 2 groups into an unrepresentative single homogenous space). The principal component analysis for the AD group is shown in the eMethods. We applied varimax rotation to promote cognitive interpretation of the emergent dimensions (as well as comparisons across the 2 multidimensional spaces). Normalized factor scores were obtained for each patient, for subsequent neuroimaging analyses and creation of the scatterplots.

**Figure 1:**
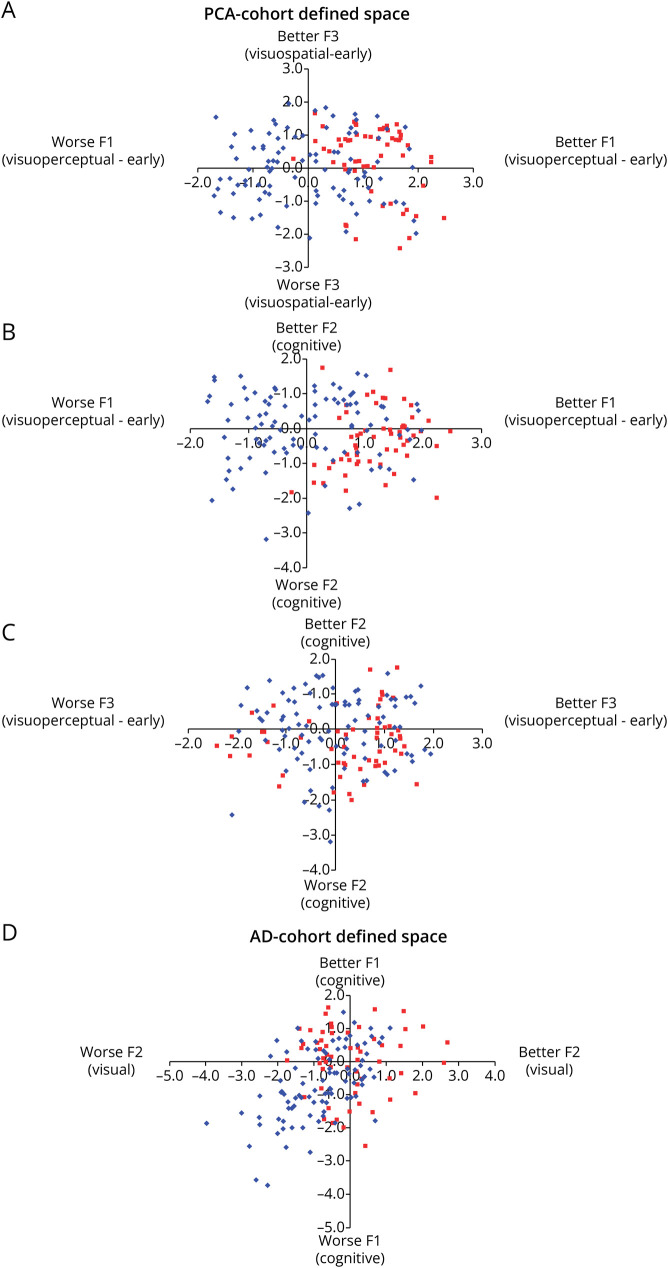
Graded Intergroup Phenotypic Variation in Posterior Cortical Atrophy and Alzheimer Disease Panels A–C: Alzheimer disease (AD) cases projected into posterior cortical atrophy (PCA) multidimensional space. Panel D: PCA cases projected into AD multidimensional space. Key: AD = red squares; PCA = blue diamonds.

Having established the multidimensional spaces for AD and PCA independently, we then explored whether there were any regions of these multidimensional spaces showing transdiagnostic overlap in impairments. This was achieved by projecting the neuropsychological scores from 1 group through the coefficient matrix of the other group (because both cohorts underwent the same cognitive test battery). The results obtained by projecting PCA patients into the AD-derived multidimensional space are presented in Figure 1, panel D (the AD principal component analysis is presented in full in the eMethods). We also explored whether the extracted components were related to disease severity (see eMethods).

#### Image Acquisition

T1-weighted volumetric MR scans were acquired for 71 healthy controls, 70 patients with PCA, and 14 patients with AD over a 10-year period from 2005 to 2015. Seven PCA and 5 AD scans were excluded after image quality assurance because of motion and ghosting artifacts, yielding a total number of 71 healthy control, 62 PCA, and 9 AD scans that were included in the final analyses. Most scans (controls: 39; PCA: 43; AD: 8) were acquired on a Siemens Prisma 3T scanner using a magnetization-prepared rapid acquisition gradient echo sequence with a 256 × 256 acquisition matrix, 282 mm field of view and the following acquisition parameters: echo time (TE) = 2.9 milliseconds, repetition time (TR) = 2,200 milliseconds, and inversion time (TI) = 900 milliseconds. The remaining images (controls: 32; PCA: 19; AD: 1) were acquired on a 1.5T Sigma MRI scanner using a spoiled gradient echo sequence with a 256 × 256 image matrix, a 240 mm field of view and the following acquisition parameters: TE = 6.3 milliseconds; TR = 14.2 milliseconds, and TI = 650 milliseconds.

#### Image Preprocessing

Image preprocessing involved the following steps conducted using Statistical Non-Parametric mapping (SnPM^31^—a toolbox within Statistical Parametric Mapping software [SPM12.1]) (1) image format conversion from Digital Imaging and Communications in Medicine to Neuroimaging Informatics Technology Initiative; (2) tissue segmentation using SPM's unified model^32^; (3) the creation of a study-specific GM segment template using SHOOT; (4) normalization of the segments to the study-specific template that generally matches standard space (Montreal Neurological Institute) in orientation using SHOOT transformations; (5) modulation to account for local volume changes; and (6) smoothing using a 6-mm full width at half-maximum Gaussian kernel to compensate for inaccuracies in spatial alignment and between-subject differences in anatomy. The smoothed, normalized and modulated SHOOT-imported GM segments were then used for analysis. Image preprocessing steps (3)–(6) were performed for the different analyses (PCA-only and Combined) separately to ensure that the GM segment template only included analysis-specific participant scans.

### Voxel-Based Morphometry

We used whole-brain VBM to explore the relationship between brain atrophy and graded variation in cognitive performance in PCA and AD. VBM analysis was performed using Statistical Non-Parametric mapping (SnPM^31^ using SPM12.1) which allows for pseudo *t* statistic images to be assessed for significance using a standard nonparametric multiple comparisons procedure based on permutation testing. Before performing the analyses, a whole-brain GM mask was defined to include only voxels for which the intensity was at least 0.2 in at least 80% of the images to circumvent exclusion of voxels most vulnerable to brain atrophy.^33^

#### Correlations Between GM Volume and Principal Component-Derived Factor Scores

Two VBM regression analyses were performed using factor scores from the PCA-derived multidimensional space, a PCA-only (N = 62) and a PCA/AD combined (N = 71) analysis to explore PCA-specific and shared PCA/AD associations between GM volume and neuropsychological deficits, respectively. The Combined VBM analysis used factor scores from the PCA principal component analysis, either directly (for PCA cases) or through projecting raw neuropsychological scores through the PCA-derived coefficient matrix (for AD cases), to relate variation in the same multidimensional space to GM volume across both groups. Both regression models included smoothed, modulated, and warped GM volume as the dependent variable, the 3 PCA principal component-generated factor scores as the independent variables, and age at assessment (mean-centered), total intracranial volume (mean-centered), sex and scanner (3T or 1.5T) as covariates. The Combined VBM analysis included group as an additional covariate. An AD-only analysis (i.e., relating GM volume to factor scores from the AD-derived multidimensional space with projected scores for PCA cases) was not performed because of the limited number of available AD scans.

Statistical significance was determined by permutation testing (10,000 permutations) based on peak-voxel inference set at *p* < 0.05 (family-wise error corrected). Scatterplots were created to visualize the relationship between GM volume and factor scores. The 3D volume results were projected to the surface using MRIcroGL (version 14).^34^

#### GM Volume Changes in PCA and AD

To aid interpretation of correlation analyses, we assessed differences in voxel-wise GM volume in PCA and AD relative to healthy controls separately using independent *t* tests. Age at assessment (mean-centered), total intracranial volume (mean-centered), sex, and scanner (3T or 1.5T) were included as covariates. Effect size maps are presented in eFigure 2.

### Data Availability

Anonymized data associated with this article will be made available by request from any qualified investigator.

## Results

### Patients

Ninety-three people with PCA and 58 people with AD were included in this study. Demographic details are summarized in Table 1. There were no significant differences between the AD and PCA groups in either age (*t*_(137)_ = 0.569, *p* = 0.571) or symptom duration (*t*_(115)_ = 1.907, *p* = 0.059). There were more female patients than male patients in the PCA group, and more male patients than female patients in the AD group (χ^2^_(1)_ = 9.35, *p* = 0.002). MMSE scores were not significantly different between AD and PCA (*t*_(141)_ = −1.73, *p* = 0.085).

**Table 2:** Neuropsychology Test Scores and Missing Data

Domain	Test	Posterior cortical atrophy		Alzheimer disease		
		Mean (SD) [min–max]	Missing data %	Mean (SD) [min–max]	Missing data %	*p* Value
Visuoperceptual	Usual views	63.5 (30.8) [0.0–100.0]	22.6	91.8 (10.4) [65.0–100.0]	13.8	<0.001^a^
	Unusual views	21.7 (23.6) [0.0–95.0]	22.6	55.4 (25.5) [5.0–95.0]	13.8	<0.001
	VOSP object decision	54.3 (22.1) [25.0–100.0]	0.0	81.6 (11.8) [55.0–100.0]	1.7	<0.001^a^
	VOSP fragmented letters	26.3 (27.8) [0.0–100.0]	11.8	71.1 (30.6) [0.0–100.0]	3.4	<0.001
Early visual	CORVIST hue discrimination	69.1 (32.7) [0.0–100.0]	8.6	81.8 (25.2) [0.0–100.0]	5.2	0.011^a^
	Crowding (time)	80.2 (21.7) [0.0–95.3]	26.9	85.1 (13.4) [0.0–93.0]	10.3	0.127^a^
	VOSP figure/ground	81.3 (15.2) [50.0–100.0]	4.3	91.8 (8.2) [70.0–100.0]	3.4	<0.001^a^
	Efron shape discrimination	74.2 (17.1) [50.0–100.0]	4.3	88.6 (15.3) [50.0–100.0]	5.2	<0.001
Episodic memory	Recognition (words)	74.4 (16.8) [48.0–100.0]	29.0	74.9 (18.8) [48.0–100.0]	36.2	0.891
	Recognition (faces)	81.9 (13.1) [48.0–100.0]	1.1	64.2 (13.3) [48.0–100.0]	34.5	<0.001
Language	Graded difficulty naming	70.5 (27.5) [0.0–100.0]	0.0	64.4 (29.6) [0.0–100.0]	0.0	0.197
	Concrete synonyms	82.1 (15.7) [0.0–100.0]	11.8	82.7 (12.4) [48.0–100.0]	15.5	0.821
	Baxter spelling	51.7 (31.9) [0.0–100.0]	6.5	58.5 (29.7) [0.0–100.0]	10.3	0.219
Executive/calculation	Graded difficulty arithmetic	45.3 (18.3) [0.0–88.5]	9.7	54.0 (19.3) [0.0–88.5]	44.8	0.027
	Digit span (forwards)	55.5 (21.3) [8.3–100.0]	23.7	52.7 (17.4) [16.7–100.0]	3.4	0.411
	Digit span (backwards)	28.3 (14.9) [0.0–100.0]	24.7	33.0 (19.9) [0.0–100.0]	1.7	0.130^a^
	Cognitive estimates	35.2 (22.4) [0.0–90.0]	4.3	34.8 (20.8) [0.0–90.0]	3.4	0.910
Visuospatial	Cancellation (N correct)	76.8 (25.7) [0.0–100.0]	3.2	95.6 (7.4) [68.4–100.0]	5.2	<0.001^a^
	Cancellation (time)	63.9 (13.9) [0.0–89.3]	5.4	52.1 (22.8) [0.0–82.2]	5.2	0.001^a^
	VOSP number location	30.4 (30.3) [0.0–100.0]	4.3	53.5 (40.2) [0.0–100.0]	5.2	<0.001^a^
	VOSP dot count (N correct)	49.2 (33.3) [0.0–100.0]	2.2	82.9 (26.3) [0.0–100.0]	5.2	<0.001^a^
	VOSP dot count (time)	74.7 (16.0) [0.0–95.7]	49.5	72.2 (18.6) [0.0–88.7]	36.2	0.523

Abbreviations: CORVIST = Cortical Vision Screening Test; N correct = number of items correct; VOSP = Visual Object and Space Perception.

Neuropsychology test scores are shown as percentage of maximum score per group (higher percentage corresponds to less impairment, less errors, faster time to complete). Missing data are shown as percentage missing per group. Significant differences between diagnostic groups on each test assessed through independent *t* tests.

a Mann-Whitney *U* statistic reported because of heterogeneity of variance.

### Neuropsychological Tests

Scores on all neuropsychological tests for AD and PCA participants are summarized in Table 2.

### Establishing the Multidimensional Spaces of PCA and AD

The principal component analysis for the PCA group was robust (Kaiser-Meyer-Olkin = 0.865) and Bartlett's test of sphericity was significant (approximate χ^2^ = 1,242.972, *df* = 231, *p* < 0.001). The 3-factor varimax rotated solution accounted for 61.0% of the total variation in the patients' performance. The variance explained per factor is as follows: Factor 1 (visuoperceptual-early) = 23.0%; Factor 2 (cognitive) = 21.4%; Factor 3 (visuospatial-early) = 16.6%. The factor loadings are shown in Table 3. A summary of tests loading onto each factor, and hence, the term used to label each factor is presented in the eMethods, with tests for the relationship of each factor with disease severity. This multidimensional space was used for the following analyses, so the principal component analysis result for the AD group alone is shown in eTable 1.

**Table 3:** Principal Component Analysis Results for Posterior Cortical Atrophy

Domain	Test	Factor 1 (visuoperceptual-early)	Factor 2 (cognitive)	Factor 3 (visuospatial-early)
Visuoperceptual	Usual views	0.894	0.070	0.244
	Unusual views	0.871	−0.037	−0.021
	VOSP object decision	0.857	0.017	0.135
	VOSP fragmented letters	0.648	0.115	0.464
Early visual	CORVIST hue discrimination	0.627	0.198	0.246
	Crowding (time)	0.655	0.222	0.429
	VOSP figure/ground	0.526	0.021	0.460
	Efron shape discrimination	0.454	0.125	0.413
Episodic memory	Recognition (words)	0.770	0.131	0.206
	Recognition (faces)	−0.098	0.548	0.258
Language	Graded Difficulty Naming	0.193	0.781	−0.036
	Concrete synonyms	0.135	0.773	0.103
	Baxter spelling	0.113	0.795	0.165
Executive/calculation	Graded Difficulty Arithmetic	0.011	0.743	0.342
	Digit span (forwards)	0.128	0.702	0.077
	Digit span (backwards)	−0.052	0.803	0.042
	Cognitive estimates	−0.202	−0.684	−0.225
Visuospatial	Cancellation (N correct)	0.365	0.305	0.589
	Cancellation (time)	0.252	0.220	0.542
	VOSP number location	0.237	0.147	0.814
	VOSP dot count (N correct)	0.163	0.037	0.823
	VOSP dot count (time)	0.186	0.318	0.648

Abbreviations: CORVIST = Cortical Vision Screening Test; N correct = number of items correct; VOSP = Visual Object and Space Perception.

### Phenotypic Continua in PCA and AD

We explored whether PCA and AD cases overlapped with each other in their respective multidimensional spaces, by projecting factor scores of 1 group into the multidimensional space of the other. AD cases (red squares) projected into the PCA-defined space are shown in Figure 1, A–C, while PCA cases (blue diamonds) projected into AD-defined space are shown in Figure 1D, also shown in eFigure 1.

These comparative plots illustrate some key observations: (1) There are graded variations along all dimensions in both patient groups; (2) there is considerable overlap between the AD and PCA groups on the general cognitive impairment dimension, irrespective of which principal component analysis solution is used; (3) the AD also overlap with the PCA group in the visuospatial and visuoperceptual dimensions extracted by the PCA cohort analysis (upper-right quadrants of Figure 1A and the right halves of B and C)—again pointing to the observation that the symptomatology of the 2 groups overlap; (4) while a subset of the PCA cases overlap with the AD cases, there are PCA cases with more pronounced visuospatial and/or visuoperceptual impairment than AD at the same level of generalized cognitive impairment.

### Shared Neural Correlates of Cognition Across Phenotypes

**Figure 2:**
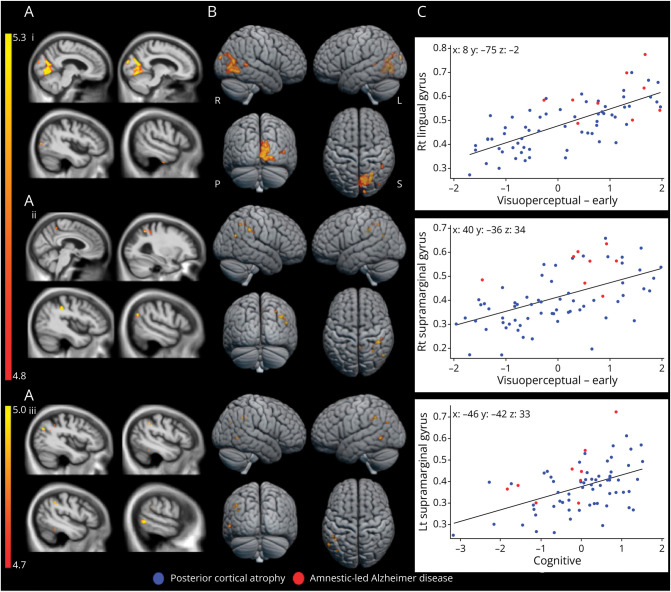
Whole-Brain VBM Results in PCA and AD Presented are significant positive associations between neuropsychological performance and GM volumes in PCA and AD. FWE-corrected significant *p* < 0.05 regions, identified by permutation-based peak-voxel inference, are shown (A) overlaid on 2-dimensional orthogonal sagittal slices of the normalized study-specific T1-weighted group average, (B) surface rendered, and (C) shows correlations between neuropsychological scores and participant-specific mean cluster GM volume values (largest significant cluster) by group as scatterplots. Color bar represents *t* values. Montreal Neurological Institute coordinates (mm) at peak voxel are shown in bold. Rt = right; Lt = left; i = visuoperceptual-early factor, ii = visuospatial-early factor and iii = cognitive factor; R = right; L = left; S = superior; P = posterior.

Regional reductions in GM volume in PCA and AD relative to control groups were consistent with previous investigations (eFigure 2). A detailed summary of the PCA VBM results can be found in eTable 2. To explore the overlapping visual and cognitive profiles in the PCA cohort multidimensional space, these profiles were related to underlying neuroanatomy in the Combined VBM. Figure 2 shows the results of this combined analysis including PCA and AD cases with available scans.

In line with the combined analysis comprising mostly PCA participant scans (PCA n = 62; AD n = 9), associations between factors and regional GM volume are broadly consistent with analyses restricted to the PCA group (see eResults). To visualize the relationship between shared neural correlates of the overlapping neuropsychological variation, Figure 2, Ci–Ciii shows, for the largest cluster associated with each principal component, the GM volume in the cluster against the corresponding factor score for every patient. This shows graded variation within and between the AD and PCA cases, for example, with several participants with AD exhibiting scores on visuoperceptual-early factors and lingual gyral atrophy which are commensurate with PCA group mean scores/atrophy. Additional correlates identified through combined analysis include lower visuospatial-early factor scores being associated with precuneal GM decreases (Table 4). These may relate to neuropsychological deficits and atrophy patterns (for example, diminished visuospatial functioning and precuneal atrophy) which are common across PCA and AD, particularly given the relatively young age of our AD sample. Overall, these results show graded, transdiagnostic phenotypic dimensions that relate to common atrophy patterns in these presentations of AD.

**Table 4:** Combined VBM Results Showing Posterior Cortical Atrophy and Alzheimer Disease Shared Brain Regions in Which GM Volume Reductions Were Associated With Lower Visuoperceptual, Visuospatial and Cognitive Factor Scores

	k	*p*_FWE_	T	x	y	z	Brain region
Visuoperceptual-early	2,038	0.0003	6.66	8	−75	−2	Right lingual gyrus
		0.0003	6.64	10	−74	20	Right intracalcarine cortex
		0.0003	6.48	11	−69	−4	Right occipital fusiform gyrus
	27	0.0014	5.99	42	−93	20	Right occipital pole
	26	0.0050	5.64	51	−80	8	Right lateral occipital cortex
	58	0.0169	5.26	36	−46	6	Right medial temporal gyrus
Visuospatial-early	120	0.0035	5.78	40	−36	34	Right supramarginal gyrus
	44	0.0020	6.02	50	−62	24	Right lateral occipital cortex
	28	0.0140	5.28	30	−54	44	Right superior parietal lobule
	21	0.0186	5.18	46	−28	30	Right parietal operculum
	17	0.0139	5.28	6	−46	50	Right precuneus
Cognitive	69	0.0051	5.56	−46	−42	33	Left supramarginal gyrus
	60	0.0067	5.46	−42	−48	38	Left angular gyrus
	60	0.0107	5.30	−56	−57	−2	Left medial temporal gyrus
	55	0.0411	4.85	−45	−46	18	Left inferior temporal gyrus
	24	0.0143	5.20	−39	−70	−28	Left lateral occipital cortex

Abbreviations: GM = grey matter; k = cluster size; PFWE = family-wise error corrected *p* value (*p* < 0.05); VBM = voxel-based morphometry; x, y, z = peak-voxel Montreal Neurological Institute coordinates.

## Discussion

The presence of AD phenotypic variations poses particular challenges for correct diagnosis and clinical management.^5,6^ This data-driven comparison of PCA and AD allowed us to consider to what extent varying presentations of AD are separable, mutually exclusive clinical categories or gradedly different positions within a single, transdiagnostic (i.e., encompassing both diagnostic groups) multidimensional space. We subsequently explored whether the cognitive impairments demonstrated in PCA and AD were associated with the same neural correlates (or could be driven by atrophy in disparate brain regions). This study provides evidence of overlapping features (visual, cognitive, and posterior cortical) in a deeply phenotyped sample of participants with PCA and AD administered the same detailed neuropsychological battery. These novel comparisons extend work investigating variation within PCA^35^ and AD,^36^ separately.

The results were broadly consistent with the conceptualization of AD and PCA as varying continuously on a spectrum of cognitive-neuroanatomical changes: (1) both AD and PCA data generated dimensions of graded and not clustered variation regarding generalized cognitive and visual impairments; (2) there was considerable overlap of the 2 patient groups along these dimensions; (3) the relationship between cognitive impairments and underlying regions of brain atrophy in PCA persisted in AD. In the remainder of the Discussion, we will consider the graded nature of the identified phenotypic variations and the implications for future clinical research and practice.

Plotting PCA and AD in the respective multidimensional space from the principal component analysis demonstrated graded variation within and between these groups with respect to visual processing impairments. As expected, a good proportion of the patients with PCA had more severe visuospatial and/or visuoperceptual impairments than the AD cases. However, there was a subset of AD cases who overlapped with PCA cases on the visual processing dimensions (Figure 1C), indicating visual deficits commensurate with mild to moderate PCA. This finding aligns with previous early reports of AD cases with pronounced visual processing deficits^2^ and recent findings suggesting a substantial proportion of patients with “typical” AD exhibit predominant visuospatial deficits.^37^ Although visual processing impairments are not necessary or sufficient for diagnosis of “typical” AD, it is generally recognized that visuospatial deficits can be present or emerge later.^38^ In our sample of amnestic-led AD cases, the profile of AD cases with visual deficits commensurate with mild to moderate PCA was not confined to AD cases with globally poor performance; some AD cases presented with impaired visual processing even when their general cognitive status was better than most other cases (top left quadrant of Figure 1C). Overall, these findings provide support for the core hypothesis for this PCA and AD comparison study, namely that both within and between presentations of AD and PCA, there is evidence of graded variation along phenotypic continua. Specifically, there is evidence of a graded dimension of visual impairment that is independent of variation in general cognitive status.

In addition to the overlap in visual processing impairments, considerable overlap of AD and PCA on the emergent “cognitive” dimensions reiterates the importance of nonvisual impairments in PCA.^4^ Others have found language deficits in early to intermediate stage PCA^39^ consistent with logopenic progressive aphasia, and there is increasing evidence of both executive deficits in PCA^40^ and frontal tau accumulation in PCA over time.^41^ The shared variations in linguistic or executive domains captured by the principal component analysis, align with a transdiagnostic reconsideration of AD and its atypical subtypes^2,10,17^ as reflecting graded involvement of different cognitive domains, rather than discrete subtypes with isolated impairments in select domains. These results also highlight the importance of fully characterizing cognitive impairments in PCA because nonvisual symptoms could contribute to the misdiagnosis of PCA.^5,7^

The results of the combined VBM analysis suggest that atrophy in the extracted clusters is associated with impairment along the extracted cognitive dimensions, regardless of diagnostic group. Neuroimaging findings imply that overlapping cognitive features in these forms of dementia may arise from atrophy in similar brain regions. This supports the conceptualization of PCA and AD as being within a shared, multidimensional phenotypic space, perhaps relating to graded neurodegeneration of functional brain networks, rather than as discrete subtypes caused by AD pathology (for a parallel proposal for the overlapping variations of logopenic progressive aphasia and AD, see the study by Ramanan et al.^17^).

Our results indicate that a simple categorical distinction between AD and PCA based on diagnostic criteria would fail to capture the evident graded differences between these phenotypes. This raises the issue of how to relate graded, multidimensional approaches to traditional, categorical classification systems.^13^ The latter provide a useful diagnostic short-hand for clinicians and may be useful for contrastive group-level analysis. We are not proposing that the diagnostic labels should be abandoned entirely. Rather, being able to place cases from different diagnostic categories into a shared, transdiagnostic multidimensional space can highlight key intra-subgroup and inter-subgroup variations, enhancing our understanding of the diagnostic categories themselves. This approach is able to capture both graded phenotypic variation, including more atypical examples and mixed cases, as well as highlight more category-like phenotypes if they are present.^14^ Thus, a comprehensive “picture” of an individual patient could include their broad label and their nuanced multidimensional profile. From a research perspective, this multidimensional approach allows for (and in fact necessitates) a more inclusive recruitment strategy which captures not only the “pure” prototypical cases but most patients, who show graded phenotypic variation.

Possible clinical ramifications include identification of (1) transdiagnostic, potentially treatable symptoms that would otherwise not be evident from research which studies only prototypical cases and (2) graded clinicoradiological dimensions also open up the possibility of new approaches to stratification of cases for treatments, dosage titration, and other elements of clinical trials research that are based on scalar rather than categorical variations.

Three methodological considerations are important to acknowledge: availability of molecular/pathologic evidence, scanner variation and sample sizes for VBM, and age of participants with AD. Although the cohorts in this study met the respective neuropsychological criteria for AD and PCA, molecular/pathologic evidence of AD was only available for a subset of cases. While all available molecular or pathologic evidence (34 PCA; 39 AD) supported underlying AD pathology and patients overall were relatively young (AD: 57 ± 6 years; PCA 60 ± 8 years), we acknowledge that we cannot rule out contributions of non-AD pathology. We do note however that for all PCA patients who have made it to autopsy (N = 11), all had a primary neuropathologic diagnosis of AD.

Regarding the VBM analysis, the imaging data were acquired on scanners of different magnetic strengths, so there is a risk that our findings could be influenced by scanner-specific factors. However, covariates for scanner were regressed out after the estimation of regional brain volumes, to separate out scanner-specific biases (over/under estimation of GM due to scanner), reducing this risk. In addition, the significantly larger proportion of scans from PCA cases (62 vs 9 scans) for combined VBM analysis could have meant that these results were driven by associations in the PCA group, which may limit generalizability.

The participants with AD were relatively young, as noted above. Patients with younger-onset AD (YOAD) can be more likely to have a predominant nonmemory impairment,^11^ which could then increase the overlap with PCA or other atypical presentations in nonmemory domains. Furthermore, YOAD has been found to have more precuneal atrophy and less pronounced medial temporal lobe atrophy compared with late-onset AD (LOAD), even in patients who show a predominant amnestic phenotype^42^; thus, YOAD cases could potentially have a parietally weighted neuroimaging profile that is more similar to PCA than LOAD. However, we also note that phenotypic heterogeneity is increasingly recognized in late-onset AD too.^37^

Taking these methodological considerations into account, we acknowledge that the results of this study represent an exploration of the shared variance in AD and PCA, as a test case for exploring the multidimensional space shared by all AD phenotypes. In future work, it will be important to confirm molecular/pathologic AD status in these cases to extend these findings towards understanding the heterogeneity caused by AD pathology specifically,^1^ to replicate these findings in larger samples (especially for VBM comparisons), and finally to replicate these findings in samples of individuals meeting criteria for LOAD to explore the potential impact of age at onset on the shared variation.

This study shows that this test-case exploration of phenotypic continua in AD and PCA has promise for uncovering the nature of variation between different clinical presentations of AD. Future research could extend this beyond amnestic-led AD and PCA, to explore (1) the full extent of variability in all clinical phenotypes associated with AD pathology and (2) variation within PCA because of different etiologies (e.g., AD, Lewy body disease, corticobasal degeneration^4^). Establishing the underpinning multidimensional space in these samples would then provide an alternative framework in which variations along each dimension (rather than differences between groups) can be related to the underpinning neuroimaging and neurobiological features.^13^ Building from situating amnestic-led AD and PCA within the same multidimensional symptom-atrophy space, future research could extend important earlier work,^2^ which captured graded differences between subgroups of neurodegenerative disease instead of comparing groups of cases based on their diagnostic label.

## Appendix Authors

**Table:**

Name	Location	Contribution
Ruth U. Ingram, PhD	Division of Psychology and Mental Health, University of Manchester, United Kingdom	Drafting/revision of the manuscript for content, including medical writing for content; study concept or design; analysis or interpretation of data
Dilek Ocal, PhD	Dementia Research Centre, UCL Institute of Neurology, London, United Kingdom	Drafting/revision of the manuscript for content, including medical writing for content; major role in the acquisition of data; analysis or interpretation of data
Ajay Halai, PhD	MRC Cognition and Brain Sciences Unit, University of Cambridge, United Kingdom	Drafting/revision of the manuscript for content, including medical writing for content; study concept or design; analysis or interpretation of data
Gorana Pobric, PhD	Division of Psychology and Mental Health, University of Manchester, United Kingdom	Drafting/revision of the manuscript for content, including medical writing for content; analysis or interpretation of data
David M. Cash, PhD	Dementia Research Centre, UCL Institute of Neurology, London, United Kingdom	Analysis or interpretation of data
Sebastian Crutch, PhD	Dementia Research Centre, UCL Institute of Neurology, London, United Kingdom	Drafting/revision of the manuscript for content, including medical writing for content; major role in the acquisition of data; study concept or design; analysis or interpretation of data
Keir X. Yong, PhD	Dementia Research Centre, UCL Institute of Neurology, London, United Kingdom	Drafting/revision of the manuscript for content, including medical writing for content; major role in the acquisition of data; study concept or design; analysis or interpretation of data
Matthew A. Lambon Ralph, PhD	MRC Cognition and Brain Sciences Unit, University of Cambridge, United Kingdom	Drafting/revision of the manuscript for content, including medical writing for content; study concept or design; analysis or interpretation of data

## Glossary

AD: Alzheimer disease
GM: grey matter
LOAD: late-onset AD
MMSE: Mini-Mental State Examination
PCA: posterior cortical atrophy
SnPM: statistical non-parametric mapping
SPM: statistical parametric mapping
TE: echo time
TI: inversion time
TR: repetition time
VBM: voxel-based morphometry
VOSP: Visual Object and Space Perception
YOAD: younger-onset AD
